# Assessment of Psychological Distress and Peer Relations among Trans Adolescents—An Examination of the Use of Gender Norms and Parent–Child Congruence of the YSR-R/CBCL-R among a Treatment-Seeking Sample

**DOI:** 10.3390/children8100864

**Published:** 2021-09-28

**Authors:** Alexandra Brecht, Sascha Bos, Laura Ries, Sibylle M. Winter, Claudia Calvano

**Affiliations:** 1 Department of Child and Adolescent Psychiatry, Charité—Universitätsmedizin Berlin, Corporate Member of Freie Universität Berlin, Humboldt Universität zu Berlin and Berlin Insitute of Health, 13353 Berlin, Germany; sascha.bos@charite.de (S.B.); lauralenaries@gmail.com (L.R.); sibylle.winter@charite.de (S.M.W.); claudia.calvano@charite.de (C.C.); 2 Department of Education and Psychology, Clinical Child and Adolescent Psychology and Psychotherapy, Freie Universität Berlin, 14195 Berlin, Germany

**Keywords:** trans adolescents, psychological distress, internalizing problems, YSR-R/CBCL-R, internalizing problems, parental congruence, peer relations, gender minority stress

## Abstract

Among trans adolescents, increased psychological distress is reported in the literature. The goal of this study was to examine psychological distress, associated peer relations and parent report congruence among the treatment-seeking sample of the Gender Identity Special Consultation (GISC) for youth at the Charité Berlin. Further, differences between the instruments’ binary gender norms were investigated. Retrospectively, we analyzed clinical data derived from the GISC. By initial interviews and using the Youth Self-Report and Child Behavior Checklist, *n* = 50 trans adolescents aged 12–18 years (*M* = 15.5) were examined for psychological problems and peer relations. Congruence between self and parent report was analyzed by correlations. Half of the sample reported suicidality, self-harm and bullying. Trans adolescents showed significantly higher internalizing and total problems than the German norm population. The congruence between self and parent report proved to be moderate to high. The level of congruence and poor peer relations were identified as predictors of internalizing problems. Significant differences between the female vs. male gender norms emerged regarding mean scores and the number of clinically significant cases. Data provide valuable implications for intervention on a peer and family level. There are limitations to the suitability of questionnaires that use binary gender norms, and further research on adequate instruments and assessment is needed.

## 1. Introduction

A topic that has been discussed, controversially, in medicine and psychology in recent years is the question of care for children and adolescents who do not identify with the gender they are assigned at birth [1]. While medicine calls this experience gender incongruence (GIC) or trans identity, affected people use various self-ascribed names to express their identity, such as transgender or genderqueer. A fluid perception between both female and male gender identity is, among others, described as gender variance. People who identify themselves neither as female nor male describe themselves, for example, as non-binary [2]. In this paper, we used the terms gender incongruence and trans as umbrella terms for people who do not identify with their at birth assigned sex (ABAS). This aims to acknowledge and include all variations of gender identity. It is noteworthy that the umbrella term used in this paper differs from the diagnosis of gender incongruence as defined in the ICD-11 classification system.

Prevalence rates for gender identity are provided by the large population-based Health Behaviour in School Aged Children study in Hamburg, Germany. Among 940 children and adolescents (10–16 years of age), 1.6% (*n* = 15) reported GIC, 1.1% gender variance and 1.5% identified themselves as non-binary [3].

Notably, treatment guidelines and legal frameworks vary across countries and even affirmative care has been of debate in several countries. In Germany, different, often interdisciplinary healthcare offers for trans adolescents, such as peer or psychological counseling, medical treatment options regarding a transition (social, judicial, medical) and endocrinological treatment, e.g., puberty-blockers, can provide time within the physical and sexual development and reduce gender dysphoria significantly [4]. In turn, a prerequisite for a medical transition is the utilization of psychological assessment and/or support.

While gender variety is a growing political and societal phenomenon—in Germany, for example, “diverse” was included as an official gender option for intersexual persons [5]—the relevance for child and adolescent psychiatry is increasing as well, as young people with GIC face several societal challenges and burdens that can lead to psychological distress. For example, in order to change their official surname, trans people have to undergo expensive psychological evaluation. Many suffer from being misgendered (being addressed as the wrong gender) and have to face discrimination, hostility and crimes [6]. Even though a tendency for increased healthcare-seeking among adolescents with GIC has been identified [7], specialized services and staff in the field of child and adolescent psychiatry are still lacking [8,9].

### 1.1. Mental Health Problems in Trans Adolescents

The prevalence of psychological distress in trans adolescents is alarmingly high. In their study among 218 adolescents aged 12–18 years with GIC and referred to the Gender Identity Development Service in London, Holt et al. [10] drew the conclusion that 49.7% suffered from depression, 23.7% from anxiety symptoms, 44% from self-harm and 39.5% from suicidal thoughts and intentions. Furthermore, 15.8% reported suicide attempts, 19.2% substance abuse, 16.4% had eating disorders, 6.8% ADHD and 5.7% psychoses. In a community-based non-clinical study on psychological distress among trans adolescents in Canada (*n* = 923) [11], 75% of the adolescents showed self-harming behavior and 65% suicidal thoughts and intentions, and 32% reported at least one suicide attempt in the past year. In Germany, too, high rates of suicidality and self-harm were found among adolescents with GIC who were referred to gender identity services [12,13]; although, thus far, surveys have been few in number and small in their sample sizes [14]. Looking at the most common psychological symptoms in adolescents with GIC, a strong tendency towards internalizing psychological problems is found [14,15]. In the area of child and adolescent psychiatry, a common way to assess mental health problems is using the questionnaires Youth Self-Report (YSR-R) and Child Behaviour Checklist (CBCL-R). There are several research groups who have used these instruments to evaluate the psychological distress of trans children and adolescents. Their findings can be summarized insofar as young trans people suffer significantly more frequently from mental health problems than their gender-conforming peer group, and especially from internalizing rather than from externalizing problems [14,16,17,18].

### 1.2. Bullying and Poor Peer Relationships

Bullying and poor peer relationships were identified as strong predictors of mental health problems in trans adolescents [10,16,19]. In the above mentioned study of Holt et al. [10], almost half of the trans adolescents (47%) experienced bullying in school and their social environment. Many young trans people suffer from social isolation and a lack of friendships and relationships [16,17]. In the literature, some research groups have investigated the association between mobbing and psychological distress using the Poor Peer Relation Scale (PPR Scale), which can be constructed out of the YSR-R. The findings showed a high level of poor peer relations among trans adolescents [16,17]. Since discrimination, mobbing and minority stress leads to mental health problems, it is important to investigate peer relations among trans adolescents who represent an often marginalized group in our society [6,19,20].

### 1.3. Gender-Related Distributions of Psychological Distress

In the general population, girls are more likely to show internalizing than externalizing problems, whereas boys are more likely to have externalizing problems [21,22,23]. The literature provides different findings regarding sex- or gender-based differences in mental health problems among trans adolescents, i.e., differences between the at birth assigned sex and the gender identity (GID). In a cross-national study of emotional and behavioral problems of trans adolescents in Toronto and Amsterdam [17], the data suggested that it was not the ABAS but the gender identity that was in line with the aforementioned general pattern. In their study, trans girls with a male at birth assigned sex (MABAS) suffered significantly more often from internalizing than externalizing problems, whereas trans boys with a female at birth assigned sex (FABAS) showed more externalizing than internalizing problems. In consequence, the authors postulated a “general pattern of reversal” [17] (p. 585) for trans adolescents since it was more common for girls to suffer from internalizing than externalizing problems and for boys vice versa. Other studies, however, found higher problem scores for trans boys [16] or no differences at all [24]. For the examination, the research group used the abovementioned YSR-R and CBCL-R. These established instruments, as well as others, evaluate the scores based on comparisons to the respective population’s gender group (boys/girls). This process tends to be unclear when working with people who cannot identify with their ABAS. Most research groups used the ABAS for the evaluation [16,17,18], whereas some others mentioned uncertainty about which YSR/CBCL gender norm should be used with trans adolescents [14,16,25]. It is still unclear how to best use these instruments when working with trans and non-binary people [25]. Rider et al. [25] investigated the influence of the selection of the gender norm male/female of the CBCL-R on the scale scores of gender-nonconforming and trans children (*n* = 55) and adolescents (*n* = 53). Differences in somatic problems, oppositional behaviour and internalizing symptoms between the binary gender-normed scores were particularly evident. However, the choice of gender norm did not appear to have a significant impact on whether a score was considered clinically significant or not. Nevertheless, whether binary-normed instruments should be used with trans adolescents is still up for debate [26,27].

### 1.4. Congruence between Parent and Child Reports on Psychological Stress in Adolescents

Another relevant issue in the use of questionnaire measures for the assessment of psychological distress in children and adolescents is the role of parental perception of distress. Firstly, the use of counseling and treatment for mental health problems in adolescents is primarily related to parental perception of psychological distress [28,29,30]. Specifically for trans adolescents, parental perception and awareness of a child’s distress play a central role with respect to their children’s coming-out process, mental health and access to medical counseling and treatment [31,32,33,34,35]. In the general population, the congruence between adolescent and parent reports on adolescent psychopathology is low to moderate [36,37,38,39]. Furthermore, among population-based and clinical non-trans samples, the discordance between the child’s and the parent’s perception of mental health problems is associated with poorer outcomes [37,40,41]. Only a few studies have investigated the congruence between parent and child report among samples of trans adolescents. For example, Zucker et al. [42] found moderate correlations between the YSR and CBCL main scales (*r* = 0.44–0.48) for adolescents with a FABAS, whereas for adolescents with a MABAS, the correlations were low to moderate and only significant for the externalizing problems scale (*r* = 0.03–0.39). In a more recent study, De Graaf [43] compared a suicidality sum score, constructed by two items from YSR-R and CBCL-R asking for suicidal ideation and intentions, between parent and child report in cross-national samples. Interestingly, the correlations differed strongly from low to strong regarding ABAS and country. Since the discordance between parent and child report was identified as a predictor for poorer health outcomes among adolescents from general samples [36,37], it is important to obtain a firm analysis about parent–child congruence among trans adolescents, and investigate if the congruence can be identified as a predictor for psychological distress, too.

### 1.5. Aims and Hypotheses 

Hence, the aims of this study are sixfold: (1) an analysis of mental health problems and gender differences among the treatment-seeking sample of trans youth, (2) the relationship between mental health problems and peer relations, (3) an evaluation of the congruence between parent and child report (4) its relation to trans adolescents’ mental health problems, and (5) investigation as to whether the reports’ congruence and poor peer relations predict psychological distress of trans adolescents. Further, we aim to (6) investigate the effect of the use of different genders norms of the YSR-/CBCL-R.

We hypothesized that (1) trans adolescents report significantly more mental health problems than the norm population, and that there are significant differences between the at birth assigned sexes (female/male) insofar as trans girls report higher internalizing problems than trans boys and trans boys report higher externalizing problems than trans girls; (2) trans adolescents’ mental health problems are positively related to poor peer relations; (3) in the self report, trans adolescents report higher levels of psychological distress than their parents in the parent report, leading to a low report congruence/correlation; (4) higher levels of incongruence are significantly positively associated with higher scores of the trans adolescents’ psychological problems; (5) the level of congruence and poor peer relations predict psychological distress significantly. We further hypothesize that (6) the results of the YSR and CBCL will be significantly different depending on the gender norm used: the gender norm for boys will lead to higher scores in internalizing problems than the norm for girls, whereas the gender norm for girls will result in higher scores in externalizing problems than the gender norm for boys.

## 2. Materials and Methods

### 2.1. Procedure

The present work investigated the psychological stress of adolescents with GIC who consulted the Gender Identity Special Consultation (GISC) at the Charité Universitätsmedizin Berlin, Germany. Affiliated with the Department of Child and Adolescent Psychiatry, the GISC is specialized in the care of children and adolescents with GIC, provided by an interdisciplinary team consisting of psychologists, endocrinologists, psychiatrists, pediatrics and speech therapists. The GISC offers interdisciplinary education and treatment services ranging from initial psychological counseling to endocrinological and phoniatric treatment. The patients seeking care are not restricted to the city of Berlin. The inclusion of caregivers as well as the recognition of the individual development stage of each person is of crucial importance. 

The GISC includes concomitant research, covering interview and questionnaire measures on gender identity, sexual orientation and mental health. Parents are asked to provide data on socioeconomic background and also to report on their child’s mental health. Further, in every initial consultation, suicidality, self-harm, other psychological symptoms or problems and bullying experiences are explored. This assessment includes the most common mental health problems in order to provide a thorough mental health screening; other parts of the psychiatric assessment such as the assessment of neurological symptoms were not included. However, in cases of a suspicion of other neurological problems, the GISC is able to refer to child psychiatrists at the clinic, which has, so far, not been the case. The diagnosis of gender incongruence according to ICD-11 is assigned and documented by the responsible psychologist according to the therapeutic assessment. This study was a retrospective analysis of the treatment-seeking sample based on the clinical documentation at time of first presentation of the GISC December 2018–November 2020. The concomitant research was approved by the Charité ethics committee (EA2/201/20, date 9 March 2020).

### 2.2. Material/Measures

#### 2.2.1. Psychological Distress

Suicidality, the occurrence of self-injuries and the experience of bullying were evaluated and documented in the initial clinical assessment session by a licensed psychologists, specialized in gender identity and variety, at the GISC. 

On a quantitative level, psychological distress was measured by the Youth Self-Report for 11–18 year olds (YSR-R) [44] and by the parent reported Child Behavior Checklist for 6–18 year olds (CBCL-R) [45]. Normative scores for the German populations are available [46]. All 120 items are answered on a 3-point scale (0 = does not apply, 1 = somewhat or sometimes applies, 2 = exactly or often applies). Both questionnaires provide eight problem scales (scales that intend to capture the construct of the respective problem area and its symptoms such as depressive/anxious), which in turn are assigned to three superordinate scales (main scales): Internalizing Problems, Externalizing Problems and Total Problems. For the assessment of clinical significance, a cut-off of a *T*-value > 69 was specified for the eight problem scales according to the manual [46]; the borderline range is from 65 to 69. For the superordinate problem scales of Internalizing and Externalizing Problems and the Total Problems scores, a *T* > 63 is clinically significant; the borderline range is defined by *T*-values from 60 to 63. The instruments’ reliability is high, given an internal consistency for the main scales of α = 0.88–0.94 for the YSR-R and α = 0.82–0.93 for the CBCL. The convergent validity is proved by high correlations with validated syndrome scales for both instruments’ scales [46]. 

Since uncertainty about the choice of gender norms when using the CBCL-R and YSR-R for young trans people had already been expressed in various studies [16,25,27], we used both female and male gender norms for each person’s evaluation. This means that the raw scores of each questionnaire were compared with the values of both boys and girls from the standardization sample, which can result in different scale scores and corresponding *T*-values.

#### 2.2.2. Peer Relations

The degree of problems in social interactions with peers is measured using three items from the YSR-R, defined as the Poor Peer Relations Scale (PPR Scale; [47]): Item 25 (“I don’t get along with other children”), Item 38 (“I get teased a lot”) and Item 48 (“I am not liked by other children”). The PPR Scale has already been used in various studies to examine adolescents with GIC [16,17,18]. In the present sample, the internal consistency of the scale is Cronbach‘s α = 0.75. A higher number of points on the PPR Scale indicates a higher degree of peer problems (range 0–6).

### 2.3. Data Analysis

Data were analyzed using SPSS 25 [48]. For the first aim, comparisons with norm scores were conducted by one-sample *t*-tests. Additionally, differences in the psychological stress between the two groups MABAS and FABAS were calculated using *t*-tests for independent samples for the scales Internalizing, Externalizing and Total Problems.

Secondly, associations between psychological distress and peer relations were analyzed by Pearson correlations. Thirdly, congruence between parent and child report was also explored by Pearson’s correlations. To calculate the difference between the self report and the parent’s report, the mean value of the CBCL-R was subtracted from the mean value of the YSR-R for each scale (DIFF = M_YSR-R − M_CBCL-R). These differences were added up to form an aggregated total difference variable (DIFF_Tot). The sign of these difference values can already provide information for each case as to whether the scale scores of the YSR-R are greater than those of the CBCL-R (positive sign) or vice versa (negative sign). In order to determine a relationship between psychological distress and parental agreement, a Pearson correlation was performed between the total difference variables and the three main scales of the YSR-R. 

For the fourth aim, we conducted a multiple linear regression analysis using the Internalizing Problem scores evaluated both by male gender norm and female gender norm as outcome variables. The predictors were entered blockwise into the regression model by entry in three steps. In the first step, the covariates age and ABAS were included in order to control possible confounding effects. In the second step, the PPR score was introduced to investigate whether poor peer relations can be identified as a predictor for psychological distress. In the third and last step, the reports’ difference variable (DIFF_Tot) according to the respective gender norm evaluation was added. Since only T-scores and no raw scores were available, we used the Internalizing Problem score as an outcome variable because the items of the PPR Scale could not be extracted from the Total Problems score, which otherwise would have led to a confounding. Based on 1000 bootstrap samples, 95% bias-corrected and accelerated confidence intervals for the coefficient *B* and its standard errors were calculated, which makes the regression robust against violations of homoscedasticity that are often found in small sample sizes as ours. For our sample size of *n* = 50 and four predictors, large effects were assumed (*f* = 0.26) which can be tested with a power of 94% (calculated by GPower).

For the fifth aim, in order to check whether the YSR-R/CBCL-R scores differ significantly depending on the use of the assigned at birth norm vs. the gender identity norm, a comparison of the mean scores of the main and problem scales of the YSR-R and CBCL-R between the male and female gender norms was carried out using the *t*-test for dependent samples. For this, only cases with the same GID (male or female) are selected. One person who identified as non-binary was excluded from these hypotheses.

Finally, it was investigated whether the number of cases with clinically increased levels of distress changed depending on the use of the assigned at birth norm versus the gender identity’s norm. For this purpose, a comparison between the dichotomous variables (0 = not clinically significant, 1 = clinically significant) according to each gender norm (cut-off value main scales: *T* > 63, problem scales: *T* > 69) (Döpfner et al., 2014), was calculated using the McNemar test. As an additional effect measure, the odds ratio (OR) was used. OR = 1 indicates no change in clinically significant proportions between evaluation according to GID and ABAS; OR < 1 means that the clinically significant cases in the evaluation according to ABAS are higher than according to GID, and OR > 1 indicates that the clinically significant proportions in the evaluation according to GID are higher than according to ABAS. Every analysis was run through a bias-corrected and accelerated (BCa) bootstrapping (α = 5%).

### 2.4. Sample

The primary inclusion criterion for this study was the determination of gender incongruence according to ICD-11 (still unpublished), a minimum age of twelve years and the presence of a complete CBCL and YSR dyad. Initially, *n* = 66 trans adolescents were referred to the GISC; *n* = 53 adolescents resp. their parents had filled out at least one questionnaire, leading to a final sample size of *n* = 50 that had filled out both YSR-R and CBCL-R. The sample was on average 15.5 years old (*SD* = 1.64; range 12–18). It consisted of 11 trans girls (22.0%) (MABAS) and 38 (76.0%) trans boys (FABAS), and one person identified as non-binary (FABAS). At this point it should be noted that some gender-variant people also use other self-descriptions and identities (e.g., non-binary, agender, queer), which, however, could not be recorded in this work and were therefore subsumed under the term trans.

## 3. Results

### 3.1. Psychological Distress

In the initial assessment interview, 54.0% of the adolescents reported suicidality (suicidal thoughts, intentions, or attempted suicide), 48.0% stated that they had already harmed themselves at least once and 44.0% had experienced bullying now or in the past.

The descriptive evaluation of YSR-R and CBCL-R shows elevated scores for the three main scales and the eight problem scales. Mean values, standard deviations and the percentage of cases whose scores are above the cut-off of the respective scale (main scale: *T* > 63, problem scale: *T* > 69) can be found in Table 1 for YSR-R and Table 2 for CBCL-R.

### 3.2. Psychological Distress of Trans Adolescents Compared to the Normative Sample

In the self-report YSR-R and in the parent report CBCL-R, the internalizing problems and total problems scales for both gender norms were significantly higher than the comparison samples, with large effect sizes (*p* < 0.001; *d* = 0.87–1.37). In contrast, no significant difference between the samples could be found for the Externalizing Problems scale in both child and parent report (*p* > 0.05) (see Table 3).

The mean values of the groups MABAS and FABAS for the main scales of the YSR-R were compared using a one-way ANOVA. No statistically significant main effect could be found between adolescents with FABAS and adolescents with MABAS with regard to their psychological distress (*p* > 0.05) (Table S1).

### 3.3. Poor Peer Relations and Mental Health Problems

Both male and female norm evaluation showed a high, significantly positive Pearson correlation between the YSR-R Internalizing Problems scale and the PPR Scale (*M* = 1.34, *SD* = 1.57, Min = 0, Max = 5) (*r* PPR ~ YSR_Int_m = 0.52 (0.22, 0.75), *p* < 0.001 and *r* PPR ~ YSR_Int_w = 0.51 (0.20, 0.73), *p* < 0.001). Thus, a statistically significant, moderate association between poor peer relationships and internalizing problems of adolescents with GIC could be found.

### 3.4. Agreement between Self and Parent Report

For the main scales of both norm evaluations, there were strong correlations between self report and parent report (*p* < 0.001). The problem scales showed moderate to strong associations between YSR-R and CBCL-R for both norm evaluations (*p* < 0.01) (Table 4).

### 3.5. Association between Psychological Distress and Parental Perception

The mean values of the difference variables according to the male norm evaluation (*M* = −1.38, *SD* = 24.81, Max = 47, Min = −52) and the female norm evaluation (*M* = −1.58, *SD* = 24.34, Max = 54, Min = −46) were both negative, i.e., the mean values of the CBCL-R were on average greater than those of the YSR-R. The relationships between the difference variables according to both norm evaluations and the Internalizing Problems and Total Problems scales turned out to be significantly positive (*p* < 0.01) with strong to moderate effects. No significant association could be found for the Externalizing Problems scale (*p* > 0.05) (Table 5). Thus, the results indicate that a larger incongruence between parental and self reports is related to a higher degree of internalizing and total problems. There were no significant differences found between the at birth assigned sexes male/female (MABAS/FABAS) regarding the level of parent–child congruence (*p* < 0.05; data not shown).

### 3.6. Poor Peer Relations and Parental Congruence as Predictors of Internalizing Problems

Table 6 provides an overview of the multiple linear regression and the results of the final model with the YSR-R Internalizing Problem scale score by male and female gender norm functioning as an outcome variable. The results of the modeling steps were: first model with age and ABAS as predictors (male norm: adjusted R^2^ = −0.01, F(2) = 1.31, *p* = 0.28; female norm: adjusted R^2^ = 0.03, F(2) = 0.1.7, *p* = 0.19) second model added the PPR score (male norm: adjusted R^2^ = 0.27; F(3) = 7.14, *p* < 0.001; female norm: adjusted R^2^ = 0.28 F(3) = 7.22, *p* < 0.001) ending with the final model introducing the reports’ difference variable (male norm: adjusted R^2^ = 0.39, F(4) = 8.75, *p* < 0.01; female norm: adjusted R^2^ = 0.36, F(4) = 0.7.84, *p* < 0.001). The overall male norm model fit was satisfactory, explaining 43.7% of the variance for the Internalizing Problem score, the female norm model was also satisfactory, explaining 41.1% of the outcome variable’s variance. The average variance of inflation (male norm: VIF = 1.11; female norm: VIF = 1.16) was not substantially greater than 1. The Durbin–Watson statistics testing the independence of errors was 2.60 (male norm) and 2.48 (female norm) and therefore within the acceptable range (1.0–3.0).

For both male and female gender norm evaluation, the PPR score and the reports’ difference variable proved to be significant predictors in the final model, whereas neither age nor ABAS turned out to be significant. Looking at the bootstrapped confidence intervals, zero lay within the intervals for the non-significant but not the significant predictors. Therefore, poor peer relations and the congruence between parent and child report could be identified as predictors for internalizing problems.

### 3.7. Differences in Assessment Results According to Gender Norm

The differences between the mean scores according to male and female norm evaluation of the main scales of the YSR-R and CBCL-R for trans boys can be seen in Table 7. For higher readability, the results for the eight problem scales of the YSR-R and CBCL-R are summarized in the Supplementary Material (Tables S1 and S2).

For trans boys, the mean values of the YSR-R main scale Internalizing Problems and the associated problem scale Physical Complaints (PC) were found to be significantly higher according to male norm evaluation than according to female norm evaluation (*p* < 0.01), with large effect sizes of *d* = 1.70 and 1.04, respectively (Table S2). Similarly, the mean score of the problem scale Rule-Breaking Behaviour (RB), proved to be significantly lower according to the male norm than according to the female norm evaluation (*p* < 0.01), with a likewise large effect size of *d* = −0.86. The mean score of the scale Thinking Problems (TP) was significantly higher according to the male norm than according to the female norm (*p* < 0.01, *d* = −0.86). No significant differences were found for the other scales (*p* > 0.05).

The CBCL-R also showed significantly higher mean values for the scale Internalizing Problems (*p* < 0.01), with a high effect size of *d* = 1.04. The mean values of the problem scale RB proved to be significantly lower according to the male norm than according to the female norm (*p* < 0.01, *d* = −0.86; Table S3). For the problem scales AP and SP, the mean score was significantly higher according to the female norm than according to the male norm (*p* < 0.05, *d* = −0.25–−0.86). No significant difference in means could be determined for the other scales. Summing up, there were significant differences between the mean values of four out of the eleven scales of the YSR-R and the CBCL-R for the evaluations according to the GID and ABAS norms for trans boys.

For trans girls, the results of the *t*-test for dependent samples between the mean scores of the main scales of the YSR-R and CBCL-R according to female and male norm evaluation are presented below (Table 8) and for the problem scales in the Supplementary Material (Tables S4 and S5). The mean scores of the YSR-R Internalizing Problems and associated problems scales (AD, WD, SC) were found to be significantly lower according to the female norm than according to the male norm (*p* < 0.01), with large effect sizes (*d* = 1.70 to −0.46); the same was found for the TP scale (*p* < 0.01, *d* = 0.69). Again, the mean values of the main and problem scales of the Externalizing Problems (RB, AB) were significantly higher according to female than male norm evaluation (*p* < 0.05, *d* = −0.35–−0.86). The mean value of the Total Problem scale proved to be significantly higher according to the male than the female norm (*p* < 0.01) and the effect size was large (*d* = 1.31). Thus, there were significant mean differences for nine scales of the YSR-R between the evaluation according to the GID and ABAS norms for trans girls.

Considering the CBCL-R, a similar pattern emerged: significantly lower mean values were found for scales of Internalizing Problems, AD, SC according to the female norm than according to the male norm (*p* < 0.05), with a large effect size (*d* = 0.67–−1.99). For the main scale Externalizing Problems, there was no significant difference (*p* > 0.05), but the mean values of the scales RB and AB as well as AP were significantly higher according to the female norm than according to the male norm (*p* < 0.05, *d* = 0.86–1.91). No significant difference in means was found for the Total Problems scale. Accordingly, significant mean differences can be found for seven scales of the CBCL-R between the evaluation according to the GID and ABAS norms for trans girls.

The results of the McNemar test between the number of clinically significant cases of YSR-R and CBCL-R are presented in each case for trans boys (Tables S5 and S6) and trans girls (Tables S7 and S8) in the Supplementary Material. Trans boys showed a significantly higher clinically significant number of cases when evaluated according to GID than according to ABAS for the Somatic Complaints (SC) scale only (*p* < 0.05); the number of significant cases of the SC scale increased by an odds ratio of 3.04 according to the male norm than according to the female norm.

Thus, in the YSR-R, a significant change in the number of clinically significant cases between the GID and ABAS norm could only be observed for trans boys for one scale. For trans girls, there was no significant change in the clinically significant number of cases between the gender norms.

Looking at the CBCL-R, there were no significant changes in the number of clinically significant cases between the norm evaluation according to GID or ABAS (*p* > 0.05), either for trans boys or for trans girls.

## 4. Discussion

This study aimed to analyze psychological distress, peer relations and the role of parental perception and norm evaluation among a healthcare-seeking sample of trans adolescents.

Regarding the first research question, our sample reported above-average scores on the Internalizing and Total Problems scale of the YSR-R and CBCL-R, with clinically significant scores of 41.3% for Internalizing and 39.7% for the Total Problems score. These high prevalence rates are in line with the findings from other studies on the accompanying psychological symptoms of adolescents with GIC [10,11,12,13]. The 44–52% rates for suicidality, self-injuries and bullying are comparable with the literature [10].

We found no differences or interactions between the male/female at birth assigned sexes in symptom scores and, therefore, no “general pattern of inversion” [17] (p. 585) with regard to a gender-related distribution of the psychological distress, which is in line with recent findings, for example, of de Graaf et al. [43]. Moreover, other studies postulated contrary results, which indicated a higher degree of internalizing and associated problems in trans boys [49,50,51]. As significantly higher levels of exposure were found in non-binary than in binary-identified trans young people [52,53], non-binary identities should also be taken into account, in addition to a binary comparison. Since only one person in the sample identified as non-binary, such a comparison could not be carried out.

Second, greater levels of poor peer relations were associated with higher levels of psychological distress and poor peer relations could be identified as a predictor for internalizing problems of trans adolescents. This is also in line with previously confirmed findings on the mental health of young trans people [10,16,17]. In terms of the Minority Stress Model [20], adolescents with GIC represent a marginalized group in heteronormative societies, which, apparently, already experiences problems with peers, a lack of friendships, exclusion and teasing in adolescence. Since trans people can also have a non-heteronormative sexual orientation, some of the young people could belong to another socially marginalized group, which could increase minority stress and the associated experience of discrimination [54,55]. Future studies with larger sample sizes are needed to obtain a deeper insight into the experiences of these subgroups.

Third, we observed moderate to high agreement between the parental and adolescent’s perception of their mental health. The data from our study showed a higher level of congruence than reported for population-based samples using the YSR and CBCL [39,56]. Additionally, the average difference between the reports was negative, implying that some parents rated psychological problems higher than their children themselves. These finding are plausible insofar as the adolescents and their parents had attended the GIF, the parents, thus, were already showing a form of support and recognition of the possible problems and the need for counseling for their child [26].

Fourth, a greater incongruity between self and parent report was associated with higher psychological stress of the adolescents, and the level of congruence could be identified as a predictor for internalizing problems. This finding is consistent with the literature on the role of parental awareness of psychological distress and supportiveness for the mental health of young trans people [32,57,58,59]. In the present study, we found no differences between the female and male at birth assigned sexes regarding the report discrepancy, which contradicts the literature on both population-based and trans samples [36,38,39,42].

Finally, following Rider et al. [25], this study examined differences in trans youth’s mental health scores between evaluation by GID and ABAS norms. This paper showed that in the YSR-R, the mean scores in the main and problem scales differed significantly on four out of eleven scales among trans boys and on nine scales among trans girls between the binary gender norms. In the CBCL-R, mean scores differed significantly on four scales for trans boys and on seven scales for trans girls. Comparable to the results by Rider et al. [25], we did not find differences between the assessment of clinical significance according to GID and ABAS, with the exception for the somatic complaints scale for trans boys in the YSR-R, which was higher after GID evaluation.

### 4.1. Limitations

With regard to our sample, it should be taken into account that the adolescents already came to our counseling with their parents and can therefore not be representative of all young trans people in Germany, nor can their parents be representative of all parents. In addition, it should be noted that in the present work, as well as in the studies described, the group of trans girls with MABAS was much smaller (*n* = 11) than that of trans boys (*n* = 48), including one non-binary person with FABAS, and mean differences and interaction effects should therefore be interpreted with caution. With regard to the comparison sample used from the CBCL-R manual, it should be noted that the validation study took place in 2001 and that the standardization may have lost its representativeness and interpretability [60]. Further, it is unclear whether this norm sample also included adolescents who did not identify with their ABAS, as gender identity was not explicitly recorded in advance [27,46]. Additionally, we were only able to run analyses by using the T-scores of each case because raw scores were not available. Using raw scores for the multiple regression could have led to more precise results and Total Problems could also have been used as an outcome variable if it was possible to extract confounding items such as the PPR scale.

### 4.2. Implications and Prospects

Reducing the high psychological distress and vulnerability of trans youth should be one of the main future advances in child and adolescent psychiatry. This may contribute to improving trans-sensitive health care in the area of child psychiatry. Future research should explore the distress’ causes. To ensure perspective, it is important to investigate in which contexts the experiences of bullying and poor peer relations are primarily reflected and to what extent it is possible for trans youth to create a supportive social network. Drawing on community-based knowledge presents as a valuable approach for professionals who offer counseling in the field of gender identity. Young trans people in particular are usually advised to network with the community, as peer counseling by other trans people or allies enables them to share experiences, to address uncertainties and victimization and to build up a social network with good peer relations. This approach is perceived as very empowering and helpful, and enables networking with other trans and genderqueer people [55]. Furthermore, awareness raising and educational measures on gender diversity should be implemented in schools to promote tolerance and openness, and to prevent discrimination in the form of bullying and bad peer relationships among both hetero- and gender-nonconforming people.

Due to the high congruence between the self and parent reports in the sample, as well as the central position of parents in the life and development of their gender-variant children, it would be relevant to explore how parents deal with their children’s trans identity and whether there might have been determining factors that led them to attend the GISC. Further research should focus on the examination of parental support, which was not assessed in our study. However, further research is needed on family and social support for trans adolescents in the context of mental health in Germany, especially from families that are yet to seek healthcare assistance, which is in line with calls from other research groups [14,16].

In the area of clinical psychology, more research should be devoted to adequate assessment tools for gender-variant young people in consultation with their parents: “To assess discrepancies between what the child desires in terms of how they want to assert their gender identity versus what the parents perceive and feel is most appropriate” [26] (p. 118). In practice with trans youth, clinicians should be aware that the decision whether to evaluate a test according to GID or ABAS has an influence on the test score but not on its interpretation, except for the somatic problems scale. Since trans people do not identify with their ABAS, it is generally questionable when the ABAS and not the GID is used for the analysis [26]. For non-binary identifying adolescents, both gender norms should be considered, although a binary-gendered test format ultimately seems rather inappropriate for this group [26]. For example, having to choose a gender when filling out the questionnaire can already raise conflicts for trans youth and their parents, triggering experiences of discrimination and building mistrust towards the investigators [25].

The results of this study emphasize that against the background of the gender-affirmative paradigm, and the avoidance of experiences of discrimination and violation, it is necessary to reconsider whether binary-normed procedures should be avoided in future. We would like to join other research groups by proposing that at least both norms should be used in the evaluation, or if a binary identification is present, the GID should be used instead of the ABAS. There are no norms for trans clients yet and validity can be raised by using both templates and deciding case by case whether the score of the female or male norm represents the person the most [25,26,27]. Since trans youth belong to a marginalized group, and appropriate measures are not available, the development of specific instruments is needed. Participatory research designs are a promising approach, giving trans adolescents and their parents a voice and concretely including them in the research process.

In practice, culturally-sensitive and gender-affirmative test diagnostics therefore require sensitivity in test selection, implementation and interpretation [27]. Thus, the results from this study underline that adolescents with GIC represent a healthcare-seeking group that requires a reflective and thinking out of the box attitude throughout the health system, and especially from professionals working in the area of child psychiatry.

## Figures and Tables

**Table 1:** Descriptive evaluation of the main and problem scales of the YSR-R (*n* = 50).

YSR Scale	Male Norm				Female Norm			
	MABAS ^12^ (*n* = 11)		FABAS ^13^ (*n* = 39)		MABAS (*n* = 11)		FABAS (*n* = 39)	
	*M*^14^ (*SD*) ^15^	% cl. Sign.^16^	*M* (*SD*)	% cl. Sign.	*M* (*SD*)	% cl. Sign.	*M* (*SD*)	% cl. Sign.
Int ^1^	62.55 (10.85)	54.5%	66.82 (12.73)	61.5%	58.27 (9.78)	54.5%	63.05 (11.78)	48.7%
Ext ^2^	52 (5.39)	-	53.59 (8.10)	12.8%	52.91 (5.77)	-	55.15 (9.18)	25.6%
Tot ^3^	59.64 (8.8)	45.5%	62.49 (10.60)	48.7%	58.55 (8.29)	45.5%	62.59 (10.30)	51.3%
AD ^4^	60.91 (10.62)	18.2%	67.05 (10.50)	43.6%	58.91 (9.24)	9.1%	64.87 (10.13)	35.9%
WD ^5^	64.18 (10.04)	18.2%	64.33 (10.39)	28.2%	62.73 (10.45)	18.2%	63.15 (10.55)	28.2%
SC ^6^	59.55 (6.65)	-	61.74 (9.98)	25.6%	56.09 (4.91)	-	58.56 (8.88)	10.3%
SP ^7^	60.82 (8.40)	18.2%	59.54 (8.33)	10.3%	60.82 (7.56)	27.3%	60.31 (8.77)	20.5%
TP ^8^	62.55 (9.22)	27.3%	67.46 (11.43)	51.3%	60.64 (8.57)	18.2%	65.90 (10.51)	48.7%
AP ^9^	59.36 (6.90)	9.1%	61.33 (11.58)	15.4%	60.18 (6.79)	18.2%	61.87 (11.40)	28.2%
RB ^10^	54.27 (6.00)	-	55.79 (5.97)	2.6%	55.27 (6.60)	9.1%	57.49 (7.16)	7.7%
AB ^11^	52.36 (2.34)	-	55.15 (5.73)	-	52.82 (2.71)	-	56.03 (6.37)	2.6%

^1^ Internalizing Problems, ^2^ Externalizing Problems, ^3^ Total Problems, ^4^ Anxious/Depressed, ^5^ Withdrawn/Depressed, ^6^ Somatic Complaints, ^7^ Social Problems, ^8^ Thought Problems, ^9^ Attention Problems, ^10^ Rule-Breaking Behaviour, ^11^ Aggressive Behaviour, ^12^ Male at birth assigned sex, ^13^ Female at birth assigned sex; ^14^ Mean; ^15^ Standard deviation; ^16^ Clinically significant (*T*-score main scale > 63, *T*-score problem scale > 69).

**Table 2:** Descriptive evaluation of the main and problem scales of the CBCL-R (*n* = 50).

CBCL Scale	Male Norm				Female Norm			
	MABAS ^12^ (*n* = 11)		FABAS ^13^ (*n* = 39)		MABAS (*n* = 11)		FABAS (*n* = 39)	
	*M*^14^ (*SD*) ^15^	% cl. Sign. ^16^	*M* (*SD)*	% cl. Sign.	*M* (*SD*)	% cl. Sign.	*M* (*SD*)	% cl. Sign.
Int ^1^	66.91 (13.10)	54.5%	66.58 (11.66)	56.4%	64.36 (12.04)	45.5%	64.13 (11.46)	56.4%
Ext ^2^	56.82 (5.81)	18.2%	54.08 (10.08)	28.2%	56.45 (6.92)	9.1%	53.59 (11.38)	25.6%
Tot ^3^	63.09 (8.14)	54.5%	61.05 (10.30)	51.3%	64.09 (8.89)	54.5%	61.77 (10.56)	53.8%
AD ^4^	63.00 (10.75)	27.3%	64.25 (10.51)	35.9%	61.82 (10.73)	27.3%	64.11 (10.70)	38.5%
WD ^5^	69.64 (14.92)	45.5%	63.35 (8.48)	12.8%	70.91 (15.17)	45.5%	64.37 (8.36)	17.9%
SC ^6^	60.55 (10.12)	27.3%	62.64 (10.30)	33.3%	58.91 (9.64)	27.3%	61.31 (10.13)	28.2%
SP ^7^	59.27 (8.91)	18.2%	58.35 (6.85)	10.3%	60.18 (10.45)	18.2%	59.36 (7.83)	17.9%
TP ^8^	60.45 (8.60)	27.3%	62.21 (8.33)	28.2%	61.00 (8.96)	27.3%	62.32 (8.38)	25.6%
AP ^9^	65.55 (11.30)	36.4%	58.30 (8.26)	10.3%	67.82 (11.14)	36.4%	59.94 (8.55)	10.3%
RB ^10^	55.45 (5.22)	-	56.05 (6.42)	5.1%	57.18 (6.42)	18.2%	57.97 (7.56)	15.4%
AB ^11^	55.73 (5.01)	-	55.02 (6.81)	5.1%	57.00 (6.29)	9.1%	56.10 (8.66)	12.8%

^1^ Internalizing Problems, ^2^ Externalizing Problems, ^3^ Total Problems, ^4^ Anxious/Depressed, ^5^ Withdrawn/Depressed, ^6^ Somatic Complaints, ^7^ Social Problems, ^8^ Thought Problems, ^9^ Attention Problems, ^10^ Rule-Breaking Behaviour, ^11^ Aggressive Behaviour, ^12^ Male at birth assigned sex, ^13^ Female at birth assigned sex; ^14^ Mean; ^15^ Standard deviation; ^16^ Clinically significant (*T*-score main scale > 63, *T*-score problem scale > 69).

**Table 3:** Results of the one-sample *t*-test between the values of the sample and the mean values of the norm sample for the main scales of the YSR-R and CBCL-R.

Variable	*M*_GISC ^6^ (*SD*) ^7^	*M*_Norm ^8^	Statistics	Effect Size ^9^	BCa 95%	
					CI+ ^10^	CI− ^10^
YSR_Int ^1^_m ^2^	65.56 (12.03)	49	*t*(53) = 10.12	*d* = 1.37 *	13.45	20.31
YSR_Ext ^3^_m	52.96 (7.44)	52	*t*(53) = 0.95	*d* = 0.16	−0.90	3.19
YSR_Tot ^4^_m	61.69 (9.76)	51	*t*(53) = 8.04	*d* = 1.08 *	8.22	13.39
YSR_Int_f ^5^	61.37 (11.47)	52	*t*(53) = 6.01	*d* = 0.87 *	6.88	12.94
YSR_Ext_f	54.09 (8.62)	52	*t*(53) = 1.78	*d* = 0.31	0.38	5.26
YSR_Tot_f	61.11 (10.01)	52	*t*(53) = 6.70	*d* = 0.97 *	7.19	12.07
CBCL_Int_m	66.82 (11.92)	54	*t*(50) = 7.69	*d* = 1.07 *	9.37	15.64
CBCL_Ext_m	54.98 (9.39)	54	*t*(51) = 0.75	*d* = 0.07	−0.21	0.35
CBCL_Tot_m	61.82 (9.87)	53	*t*(50) = 6.39	*d* = 0.87 *	5.84	11.13
CBCL_Int_f	64.11 (11.97)	53	*t*(53) = 6.82	*d* = 0.96 *	7.77	14.37
CBCL_Ext_f	54.31 (10.84)	52	*t*(53) = 1.57	*d* = 0.21	−0.79	5.10
CBCL_Tot_f	62.41 (10.52)	53	*t*(53) = 6.57	*d* = 0.89 *	6.52	11.90

^1^ Internalizing Problems; ^2^ Male norm; ^3^ Externalizing Problems; ^4^ Total problems; ^5^ Female norm; ^6^ Mean of the GISC-sample; ^7^ Standard deviation; ^8^ Mean of the manual’s norm sample; ^9^ Significance and effect; * *p* < 0.001; ^10^ 95% bias-corrected and accelerated confidence interval of mean differences. Each mean of the main scale’s score in YSR-R and CBCL-R of the current sample was compared with the respective mean score of the manual’s norm population score using both male and female gender norms.

**Table 4:** Correlations between main and problem scales of the CBCL-R and YSR-R according to male norm evaluation.

Scale	*r* YSR_CBCL_m ^12^	BCa 95% CI ^14^	*r* YSR_CBCL_f ^13^	BCa 95% CI ^14^
Int ^1^	0.59 **	(0.14, 0.58)	0.61 *	(0.42, 0.76)
Ext ^2^	0.51 **	(0.19, 0.76)	0.61 *	(0.40, 0.76)
Tot ^3^	0.57 **	(0.34, 0.74)	0.60 *	(0.39, 0.74)
AD ^4^	0.47 **	(0.22, 0.70)	0.55 *	(0.31, 0.76)
WD ^5^	0.47 **	(0.25, 0.70)	0.45 *	(0.21, 0.69)
SC ^6^	0.56 **	(0.29, 0.74)	0.52 *	(0.27, 0.70)
SP ^7^	0.44 **	(0.16, 0.66)	0.48 *	(0.23, 0.66)
TP ^8^	0.53 **	(0.31, 0.71)	0.54 *	(0.35, 0.71)
AP ^9^	0.40 **	(0.22, 0.61)	0.45 *	(0.25, 0.65)
RB ^10^	0.53 **	(0.25, 0.72)	0.65 *	(0.43, 0.79)
AB ^11^	0.41 **	(0.11, 0.67)	0.49 *	(0.21, 0.71)

^1^ Internalizing Problems, ^2^ Externalizing Problems, ^3^ Total Problems, ^4^ Anxious/Depressed, ^5^ Withdrawn/Depressed, ^6^ Somatic Complaints, ^7^ Social Problems, ^8^ Thought Problems, ^9^ Attention Problems, ^10^ Rule-Breaking Behaviour, ^11^ Aggressive Behaviour, ^12^ Male norm, ^13^ Female norm; * *p* < 0.01, ** *p* < 0.001; ^14^ 95% bias-corrected and accelerated confidence interval of the correlation.

**Table 5:** Correlations and 95%-confidence intervals between the difference variable according to male and female norm evaluation with the corresponding scores of the main scales of the YSR-R.

	Int ^5^_m/f	Ext ^6^_m/f	Tot ^2^_m/f
DIFF ^1^_Tot ^2^_m ^3^	0.49 ** (0.24, 0.67)	0.23 (−0.04, 0.46)	0.43 ** (0.20, 0.62)
DIFF_Tot_f ^4^	0.47 ** (0.25, 0.67)	0.26 (0.04, 0.47)	0.39 * (0.18, 0.58)

^1^ Difference variable of the report’s congruence; ^2^ Total Problems; ^3^ Male norm; ^4^ Female norm; ^5^ Internalizing Problems; ^6^ Externalizing Problems; * *p* < 0.05; ** *p* < 0.01.

**Table 6:** Final linear model of predictors of the YSR-R Internalizing Problem score evaluated by male and female gender norm, with 95% bias-corrected and accelerated confidence intervals reported in square brackets. Confidence intervals and standard errors based on 1000 bootstrap samples.

	Male Norm				Female Norm			
	Unstandardized Coefficients		Standardized Coefficents		Unstandardized Coefficients		Standardized Coefficents	
	B ^5^	SE ^6^ B	β	*p*	B	SE B	β	*p*
Constant	53.33 (30.07, 79.94)	13.18		*p* < 0.01	49.69 (24.08, 77.16)	12.43		*p* < 0.01
ABAS ^1^	1.62 (−4.88, 7.19)	3.18	0.06	*p* = 0.64	2.01 (−4.83, 7.44)	3.06	0.07	*p* = 0.55
Age ^2^	0.44 (−1.29, 2.08)	0.82	0.06	*p* = 0.62	0.43 (−1.05, 1.88)	0.78	0.06	*p* = 0.62
PPR score ^3^	3.54 (1.96, 5.17)	0.78	0.45	*p* < 0.001	3.19 (1.70, 4.86)	0.75	0.44	*p* < 0.01
DIFF score ^4^	0.19 (0.08, 0.30)	0.06	0.38	*p* < 0.01	0.16 (0.05, 2.7)	0.06	0.35	*p* < 0.05

^1^ At birth assigned sex; ^2^ Age at initial session; ^3^ Poor Peer Relations Scale score; ^4^ Difference between parent and child report; ^5^ Regression coefficient; ^6^ Standard error.

**Table 7:** Differences between the gender norms for the main scales of YSR-R and CBCL-R.

Trans Boys (*n* = 38)						
Scale	Male Norm	Female Norm		BCa 95%		
	*M*_GID ^4^ (*SD*) ^5^	*M*_ABAS ^6^ (*SD*)	Statistics	Effect Size	CI+ ^7^	CI− ^7^
YSR_Int ^1^	66.21 (12.31)	62.45 (11.30)	*t*(37) = −6.42	*d* = 1.04 *	2.20	4.63
YSR_Ex ^2^	53.76 (8.14)	55.34 (9.23)	*t*(37) = −3.89	*d* = −0.63	−2.57	−0.97
YSR_Tot ^3^	62.24 (10.62)	62.39 (10.36)	*t*(37) = −0.18	*d* = −0.03	−2.38	1.25
CBCL_Int	66.55 (11.81)	64.08 (11.61)	*t*(37) = −5.54	*d* = 1.04 *	−3.32	−1.62
CBCL_Ext	54.40 (10.02)	53.87 (11.39)	*t*(37) = −0.49	*d* = −0.63	−2.76	1.31
CBCL_Tot	61.08 (10.43)	61.79 (10.70)	*t*(37) = 1.27	*d* = −0.03	−0.40	1.68

^1^ Internalizing Problems; ^2^ Externalizing Problems; ^3^ Total Problems; ^4^ Mean according gender identity norm; ^5^ Standard deviation; ^6^ Mean according at birth assigned sex norm; ^7^ 95% bias-corrected and accelerated confidence interval for mean differences; * *p* < 0.01.

**Table 8:** Differences between the gender norms for the main scales of YSR-R and CBCL-R for trans girls.

Trans Girls (*n* = 11)						
Variable	Female Norm	Male Norm			BCa 95%	
	*M*_GID (SD) ^4^	*M*_ABAS (SD) ^5^	Statistics	Effect Size	CI+ ^6^	CI− ^6^
YSR_Int ^1^	58.27 (9.78)	62.55 (10.85)	*t*(10) = 12.84	*d* = 1.04 *	3.60	4.90
YSR_Ext ^2^	52.91 (5.77)	52.00 (5.39)	*t*(10) = −4.30	*d* = −0.63 *	−1.30	−0.42
YSR_Tot ^3^	58.55 (8.29)	59.64 (8.08)	*t*(10) = 4.35	*d* = 1.31 *	0.57	1.56
CBCL_Int	64.36 (12.04)	66.91 (13.10)	*t*(10) = 12.84	*d* = −1.99 *	−3.37	−1.76
CBCL_Ext	56.45 (6.92)	56.82 (5.81)	*t*(10) = −4.30	*d* = −0.05	−6.11	2.86
CBCL_Tot	64.09 (8.89)	63.09 (8.14)	*t*(10) = 4.35	*d* = −0.03	0.22	1.75

^1^ Internalizing Problems; ^2^ Externalizing Problems; ^3^ Total Problems; ^4^ Mean according gender identity norm; ^5^ Mean according at birth assigned sex norm; ^6^ 95% bias-corrected and accelerated confidence interval for mean differences; * *p* < 0.01.

## Data Availability

The data presented in this study are available on appropriate request from the corresponding author. The data are not publicly available as the privacy of the human subjects must be ensured.

## Supplementary Materials

The following are available online at https://www.mdpi.com/article/10.3390/children8100864/s1. Table S1. Results of the one-way ANOVA for testing the effect of the at birth assigned sex on the main problem scale scores of the YSR-R; Table S2. Results of the *t*-test for dependent samples between means according to gender norms of the main and problem scales of the YSR-R for trans boys; Table S3. Results of the *t*-test for dependent samples between means according to gender norms of the main and problem scales of the CBCL-R for trans boys; Table S4. Results of the *t*-test for dependent samples between means according to gender norms of the main and problem scales of the YSR-R for trans girls; Table S5. Results of the *t*-test for dependent samples between means according to gender norms of the main and problem scales of the CBCL-R for trans girls; Table S6. Results of the McNemar test indicating the change between the clinical significance of the evaluation norms from GID to ABAS from the YSR-R for trans boys; Table S7. Results of the McNemar test indicating the change between the clinical significance of the evaluation norms from GID to ABAS from the CBCL-R for trans girls; Table S8. Results of the McNemar test indicating the change between the clinical significance of the evaluation norms from GID to ABAS from the YSR-R for trans girls; Table S9. Results of the McNemar test indicating the change between the clinical significance of the evaluation norms from GID to ABAS from the CBCL-R for trans girls.
