# Supplementary material for: Assessment of Psychological Distress and Peer Relations among Trans Adolescents—An Examination of the Use of Gender Norms and Parent–Child Congruence of the YSR-R/CBCL-R among a Treatment-Seeking Sample
Source: Children (Basel). 2021 Sep 28;8(10):864. doi: 10.3390/children8100864 (PMC8535001; doi:10.3390/children8100864)
Supplement: Supplementary file 1 [file children-08-00864-s001.zip › children-1369223-supplementary.pdf]

children

MDPI

Supplementary Materials

Table S1. Results of the one-way ANOVA for testing the effect of the at birth assigned sex on the main problem scale scores of the YSR-R.

	**Male norm**		**Female norm**	
Scale	Statistics	Sig., Effect size[4]	Statistics	Sig., Effect size
Int[1]	$F(1, 48) = 1.03$	$p = .32$, $d = .36$	$F(1, 48) = 1.51$	$p = .23$, $d = .43$
Ext[2]	$F\ (1, 48) = .37$	$p = .54$, $d = .21$	$F(1, 48) = .59$	$p = .45$, $d = .27$
Tot[3]	$F(1, 48) = .68$	$p = .41$, $d = .29$	$F(1, 48) = .1.43$	$p = .24$, $d = .42$

[1]Internalizing Problems; [2]Externalizing Problems; [3]Total Problems; [4]Significance and effect size.

Table S2. Results of the t-test for dependent samples between means according to gender norms of the main and problem scales of the YSR-R for trans boys.

**trans boys (n = 38)**						
		**Male norm**	**Female norm**		**BCa 95%**	
Scale	***M*_GID (*SD*)**[12]	***M*_ABAS (*SD*)**[13]	**Statistics**[14]	**Sig., Effect**[15]	**CI +**[16]	**CI -**[16]
YSR_Int[1]	66.21 (12.31)	62.45 (11.30)	$t(37) = -6.42$	$p < .01$, $d = 1.04$	2.20	4.63
YSR_Ext[2]	53.76 (8.14)	55.34 (9.23)	$t(37) = -3.89$	$p = .135$, $d = -.63$	-2.57	-.97
YSR_Ges[3]	62.24 (10.62)	62.39 (10.36)	$t(37) = -.18$	$p = .818$, $d = -.03$	-2.38	1.25
YSR_AD[4]	66.39 (9.79)	64.18 (9.30)	$t(37) = 2.82$	$p = 1.83$, $d = -.46$	.31	3.40
YSR_WD[5]	63.63 (9.55)	62.42 (9.64)	$t(37) = 3.68$	$p = .057$, $d = -.60$	.351	1.72
YSR_SC[6]	61.58 (10.06)	58.45 (8.96)	$t(37) = 10.45$	$p < .01$, $d = 1.70$	2.59	3.59
YSR_SP[7]	59.34 (8.35)	60.08 (8.77)	$t(37) = -1.57$	$p = .327$, $d = -.25$	-2.04	.00
YSR_TP[8]	67.18 (11.45)	65.63 (10.51)	$t(37) = 4.25$	$p < .01$, $d = -.69$	.71	2.17
YSR_AP[9]	61.24 (11.72)	61.76 (11.53)	$t(37) = -1.16$	$p = .385$, $d = -.19$	-1.64	.166
YSR_RB[10]	55.95 (5.97)	57.68 (7.14)	$t(37) = -5.33$	$p < .01$, $d = -.86$	-2.57	-1.15
YSR_AB[11]	55.29 (5.75)	56.18 (6.38)	$t(37) = -2.13$	$p = .37$, $d = -.35$	-1.97	-.32

[1]Internalizing problems; [2]Externalizing Problems; [3]Total Problems; [4]Anxious/Depressed; [5]Withdrawn/Depressed; [6]Somatic Complaints; [7]Social Problems; [8]Thought Problems; [9]Attention Problems [10]Rule-Breaking Behavior; [11]Aggressive Behavior; [12]Mean according to gender identity norm; [13]Mean according to at birth assigned sex norm[14]Statistics of t-test for dependent samples; [15]Significance and effects; [16]95% bias-corrected and accelerated confidence interval.

Table S3. Results of the t-test for dependent samples between means according to gender norms of the main and problem scales of the CBCL-R for trans boys.

trans boys (n = 38)						
	**Male norm**	**Female norm**		**BCa 95%**		
Variable	***M*_GID (*SD*)**[12]	***M*_ABAS (SD)**[13]	**Statistics**[14]	**Sig., Effect**[15]	**CI +**[16]	**CI -**[16]
CBCL _Int[1]	66.55 (11.81)	64.08 (11.61)	$t(37) = -5.54$	$p < .01$, $d = 1.04$	-3.32	-1.62
CBCL _Ext[2]	54.40 (10.02)	53.87 (11.39)	$t(37) = -.49$	$p = .627$, $d = -.63$	-2.76	1.31
CBCL _Ges[3]	61.08 (10.43)	61.79 (10.70)	$t(37) = 1.27$	$p = .250$, $d = -.03$	-.40	1.68
CBCL _AD[4]	64.23 (10.65)	64.12 (10.84)	$t(37) = -.15$	$p = 867$, $d = -.46$	-1.32	1.30
CBCL _WD[5]	63.36 (8.60)	64.35 (8.47)	t(37) = 1.95	$p = .146$, $d = -.60$	.27	1.82
CBCL _SC[6]	62.52 (10.42)	61.24 (10.26)	$t(37) = -1.97$	$p = .172$, $d = 1.70$	-2.27	-.04
CBCL _SP[7]	58.26 (6.91)	59.24 (7.90)	$t(37) = 2.28$	$p < .05$, $d = -.25$	.113	1.82
CBCL _TP[8]	62.14 (8.43)	62.24 (8.48)	$t(37) = .25$	$p = .830$, $d = -.69$	-.82	.81
CBCL _AP[9]	58.28 (8.37)	59.86 (8.65)	$t(37) = 3.29$	$p < .01$, $d = -.19$	.64	2.35
CBCL _RB[10]	56.20 (6.42)	58.12 (7.60)	$t(37) = 5.45$	$p < .01$, $d = -.86$	1.25	2.56
CBCL _AB[11]	55.15 (6.85)	56.26 (8.72)	$t(37) = 1.74$	$p = .084$, $d = -.35$	-.15	2.19

[1]Internalizing Problems; [2]Externalizing Problems; [3]Total Problems; [4]Anxious/Depressed; [5]Withdrawn/Depressed; [6]Somatic Complaints; [7]Social Problems; [8]Thought Problems; [9]Attention Problems [10]Rule-Breaking Behavior; [11]Aggressive Behavior; [12]Mean according to gender identity norm; [13]Mean according to at birth assigned sex norm[14]Statistics of t-test for dependent samples; [15]Significance and effects; [16]95% bias-corrected and accelerated confidence interval.

Table S4. Results of the t-test for dependent samples between means according to gender norms of the main and problem scales of the YSR-R for trans girls.

trans girls (n = 11)						
	**Female norm**	**Male norm**		**BCa 95%**		
Scale	***M*_GID (*SD*)**[12]	***M*_ABAS (*SD*)**[13]	**Statistics**[14]	**Sig., Effect**[15]	**CI +**[16]	**CI -**[16]
YSR_Int[1]	58.27 (9.78)	62.55 (10.85)	$t(10) = 12.84$	$p < .01$, $d = 1.04$	3.60	4.90
YSR_Ext[2]	52.91 (5.77)	52.00 (5.39)	$t(10) = -4.30$	$p < .01$, $d = -.63$	-1.30	-.42
YSR_Ges[3]	58.55 (8.29)	59.64 (8.08)	$t(10) = 4.35$	$p < .01$, $d = 1.31$	.57	1.56
YSR_AD[4]	58.91 (9.24)	60.91 (10.62)	$t(10) = 3.96$	$p < .05$, $d = -.46$	1.11	2.83

YSR_WD[5]	62.73 (10.45)	64.18 (10.04)	$t(10) = 5.16$	$p < .01$, $d = -.60$	.88	2.00
YSR_SC[6]	56.09 (4.91)	59.55 (6.65)	$t(10) = 5.98$	$p < .05$, $d = 1.70$	2.31	4.50
YSR_SP[7]	60.82 (8.40)	60.82 (7.56)	$t(10) = .00$	$p = 1.00$, $d = -.25$	-1.23	1.59
YSR_TP[8]	60.64 (8.57)	62.55 (9.22)	$t(10) = 4.87$	$p < 01$, $d = -.69$	1.14	2.75
YSR_AP[9]	60.18 (6.79)	59.36 (6.90)	$t(10) = -1.94$	$p = .084$, $d = -.19$	-1.58	.00
YSR_RB[10]	55.27 (6.60)	54.27 (5.67)	$t(10) = -3.71$	$p < .01$, $d = -.86$	-1.60	-.50
YSR_AB[11]	52.82 (2.72)	52.36 (2.34)	$t(10) = -2.89$	$p < .05$, $d = -.35$	-.78	-.17

[1]Internalizing problems; [2]Externalizing problems; [3]Total problems; [4]Anxious/depressed; [5]Withdrawn/Depressed; [6]Somatic Complaints; [7]Social Problems; [8]Thought Problems; [9]Attention Problems [10]Rule-Breaking Behavior; [11]Aggressive Behavior; [12]Mean according to gender identity norm; [13]Mean according to at birth assigned sex norm[14]Statistics of t-test for dependent samples; [15]Significance and effects; [16]95% bias-corrected and accelerated confidence interval.

Table S5. Results of the t-test for dependent samples between means according to gender norms of the main and problem scales of the CBCL-R for trans girls.

		**trans girls (*n* = 11)**				
	**Female norm**	**Male Norm**		**BCa 95%**		
**Scale**	***M*_GID (*SD*)[12]**	***M*_ABAS (*SD*)[13]**	**Statistics[14]**	**Sig., Effect[15]**	**CI +[16]**	**CI -[16]**
**CBCL _Int[1]**	64.36 (12.04)	66.91 (13.10)	$t(10)= 12.84$	$p < .01$, $d = -1.99$	-3.37	-1.76
**CBCL _Ext[2]**	56.45 (6.92)	56.82 (5.81)	$t(10) = -4.30$	$p = .852$, $d = -.05$	-6.11	2.8
**CBCL _Ges[3]**	64.09 (8.89)	63.09 (8.14)	$t(10) = 4.35$	$p = .080$, $d = -.03$	.22	1.75
**CBCL _AD[4]**	61.82 (10.73)	63.00 (10.75)	$t(10) = 3.96$	$p < .01$, $d = .67$	-1.56	-.80
**CBCL _WD[5]**	70.91 (15.17)	69.64 (14.92)	$t(10) = 5.16$	$p < .01$, $d = 1.15$	.53	1.91
**CBCL _SC[6]**	58.91 (9.64)	60.55 (10.12)	$t(10) = 5.98$	$p < .05$, $d = -1.00$	-2.83	-.55
**CBCL _SP[7]**	60.18 (10.45)	59.27 (8.91)	$t(10) = .00$	$p = .114$, $d = .54$	-.10	1.91
**CBCL _TP[8]**	61.00 (8.96)	60.45 (8.60)	$t(10) = 4.87$	$p = .237$, $d = .45$	.09	1.40
**CBCL _AP[9]**	67.82 (11.14)	65.55 (11.30)	$t(10) = -1.94$	$p < .01$, $d = 1.91$	1.56	2.93
**CBCL _RB[10]**	57.18 (6.42)	55.45 (5.22)	$t(10) = -3.71$	$p < .05$, $d = .91$	.58	2.67
**CBCL _AB[11]**	57.00 (6.29)	55.73 (5.10)	$t(10) = -2.89$	$p < .05$, $d = .86$	.46	2.14

[1]Internalizing Problems; [2]Externalizing Problems; [3]Total Problems; [4]Anxious/Depressed; [5]Withdrawn/Depressed; [6]Somatic Complaints; [7]Social Problems; [8]Thought Problems; [9]Attention Problems [10]Rule-Breaking Behavior; [11]Aggressive Behavior; [12]Mean according to gender identity norm; [13]Mean according to at birth assigned

sex norm;[14]Statistics of t-test for dependent samples; [15]Significance and effects; [16]95% bias-corrected and accelerated confidence interval of mean differences.

Table S6. Results of the McNemar-Test indicating the change between the clinical significance of the evaluation norms from GID to ABAS from the YSR-R for trans boys.

trans boys (n = 38)						
	**Male norm**	**Female norm**	**BCa 95%**			
**Scale**	***N*_cl. sign. (%)[12]**	***N*_cl. sign. (%)[13]**	**Sig. McNemar[14]**	**OR[15]**	**CI+[16]**	**CI-[16]**
**YSR_Int[1]**	23 (60.5)	18 (47.4)	p = .125	1.7	0.69	4.23
**YSR_Ext[2]**	5 (13.2)	10 (26.3)	p = .063	0.42	0.13	1.39
**YSR_Ges[3]**	18 (47.4)	19 (50.0)	p = 1.000	0.9	0.37	2.21
**YSR_AD[4]**	16 (42.1)	11 (28.9)	p = .125	1.79	0.69	4.62
**YSR_WD[5]**	10 (26.3)	6 (15.8)	p = .125	1.9	0.61	5.91
**YSR_SC[6]**	10 (26.3)	4 (10.5)	p < .05	3.04	0.86	10.73
**YSR_SP[7]**	3 (7.9)	4 (10.5)	p = 1.000	0.73	0.15	3.5
**YSR_TP[8]**	18 (47.4)	15 (39.5)	p = .250	1.38	0.56	3.43
**YSR_AP[9]**	6 (15.8)	6 (15.8)	p = 1.000	1	0.29	3.43
**YSR_RB[10]**	1 (2.6)	2 (5.3)	p = 1.000	0.49	0.04	5.6
**YSR_AB[11]**	—	—	—	—		

[1]Internalizing Problems; [2]Externalizing Problems; [3]Total Problems; [4]Anxious/Depressed; [5]Withdrawn/Depressed; [6]Somatic Complaints; [7]Social Problems; [8]Thought Problems; [9]Attention Problems [10]Rule-Breaking Behavior; [11]Aggressive Behavior; [12]Number/percentage of clinical significant cases (T > 63/69) according gender identity norm; [13]Number/percentage of clinical significant cases according at birth assigned sex norm; [14]Significance of McNemar-Test; [15]Odds ratio; [16]95% bias-corrected and accelerated confidence interval.

Table S7. Results of the McNemar-Test indicating the change between the clinical significance of the evaluation norms from GID to ABAS from the CBCL-R for trans girls.

trans boys (n = 38)						
	**Male norm**	**Female norm**	**BCa 95%**			
**Scale**	***N*_cl.sign. (%)[12]**	***N*_cl.sign. (%)[13]**	**Sig. McNemar[14]**	**OR[15]**	**CI+[16]**	**CI-[16]**
**CBCL_Int[1]**	20 (52.6)	21 (55.3)	p = 1.000	0.9	0.36	2.22
**CBCL_Ext[2]**	11 (29.0)	10 (26.3)	p = 1.000	1.14	0.42	3.12

**CBCL_Ges**[3]	20 (52.6)	21 (55.3)	$p = 1.000$	0.9	0.36	2.22
**CBCL_AD**[4]	14 (36.8)	11 (28.9)	$p = .375$	1.43	0.55	3.75
**CBCL_WD**[5]	5 (13.2)	5 (13.2)	$p = 1.000$	1	0.26	3.78
**CBCL_SC**[6]	11 (29.0)	7 (18.4)	$p = .219$	1.8	0.61	5.31
**CBCL_SP**[7]	2 (5.3)	4 (10.5)	$p = 6.25$	0.47	0.08	2.75
**CBCL_TP**[8]	7 (18.4)	10 (26.3)	$p = .375$	0.63	0.21	1.89
**CBCL_AP**[9]	4 (10.5)	4 (10.5)	$p = 1.000$	1	0.23	4.33
**CBCL_RB**[10]	1 (2.6)	4 (10.5)	$p = .250$	0.23	0.02	2.16
**CBCL_AB**[11]	2 (5.3)	5 (13.2)	$p = .375$	0.37	0.37	2.02

[1]Internalizing Problems; [2]Externalizing Problems; [3]Total Problems; [4]Anxious/Depressed; [5]Withdrawn/Depressed; [6]Somatic Complaints; [7]Social Problems; [8]Thought Problems; [9]Attention Problems [10]Rule-Breaking Behavior; [11]Aggressive Behavior; [12]Number/percentage of clinical significant cases ($T > 63/69$) according gender identity norm; [13]Number/percentage of clinical significant cases according at birth assigned sex norm; [14]Significance of McNemar-Test; [15]Odds ratio; [16]95% bias-corrected and accelerated confidence interval.

Table S8. Results of the McNemar-Test indicating the change between the clinical significance of the evaluation norms from GID to ABAS from the YSR-R for trans girls.

**trans girls (*n* = 11)**						
	**Female norm**	**Male norm**		**BCa 95%**		
Scale	***N*_cl. sign. (%)**[12]	***N*_cl. sign. (%)**[13]	**Sig. McNemar**[14]	**OR**[15]	**CI+**[16]	**CI-**[16]
YSR_Int[1]	6 (54.5)	6 (54.5)	$p = 1.000$	1	0.19	5.36
YSR_Ext[2]	—	—	—	—		
YSR_Ges[3]	5 (45.5)	5 (45.5)	$p = 1.000$	1	0.19	5.36
YSR_AD[4]	1 (9.1)	2 (18.2)	$p = 1.000$	0.45	0.03	5.84
YSR_WD[5]	1 (9.1)	2 (18.2)	$p = 1.000$	0.45	0.03	5.84
YSR_SC[6]	—	—	—	—		
YSR_SP[7]	1 (9.1)	1 (9.1)	$p = 1.000$	1	0.05	18.3
YSR_TP[8]	2 (18.2)	3 (27.3)	$p = 1.000$	0.59	0.08	4.5
YSR_AP[9]	1 (9.1)	1 (9.1)	$p = 1.000$	1	0.05	18.3
YSR_RB[10]	1 (8.1)	—	—	—		

YSR_AB[11]	—	—	—	—

[1]Internalizing Problems; [2]Externalizing Problems; [3]Total Problems; [4]Anxious/Depressed; [5]Withdrawn/Depressed; [6]Somatic Complaints; [7]Social Problems; [8]Thought Problems; [9]Attention Problems [10]Rule-Breaking Behavior; [11]Aggressive Behavior; [12]Number/percentage of clinical significant cases ($T > 63/69$) according gender identity norm; [13]Number/percentage of clinical significant cases according at birth assigned sex norm; [14]Significance of McNemar-Test; [15]Odds ratio; [16]95% bias-corrected and accelerated confidence interval.

Table S9. Results of the McNemar-Test indicating the change between the clinical significance of the evaluation norms from GID to ABAS from the CBCL-R for trans girls.

**trans* Mädchen (*n* = 11)**						
	**Female norm**	**Male norm**		**BCa 95%**		
Scale	***N*_cl.auff. (%)[12]**	***N*_ cl.sign. (%)[13]**	**Sig. McNemar[14]**	**OR[15]**	**CI+[16]**	**CI-[16]**
CBCL_Int[1]	5 (45.5)	6 (54.5)	$p = 1.000$	0.69	0.13	3.72
CBCL_Ext[2]	1 (9.1)	2 (18.2)	$p = 1.000$	0.45	0.03	5.84
CBCL_Ges[3]	6 (54.5)	6 (54.5)	$p = 1.000$	1	0.19	5.36
CBCL_AD[4]	3 (27.3)	3 (27.3)	$p = 1.000$	1	0.15	6.53
CBCL_WD[5]	5 (45.5)	5 (45.5)	$p = 1.000$	0.45	0.08	2.67
CBCL_SC[6]	3 (27.3)	3 (27.3)	$p = 1.000$	1	0.15	6.53
CBCL_SP[7]	2 (18.2)	2 (18.2)	$p = 1.000$	1	0.11	8.73
CBCL_TP[8]	3 (27.3)	2 (18.2)	$p = 1.000$	1.69	0.22	12.81
CBCL_AP[9]	4 (36.4)	4 (36.4)	$p = 1.000$	1	0.18	5.68
CBCL_RB[10]	—	—	—	—		
CBCL_AB[11]	1 (9.1)	—	—	—		

[1]Internalizing Problems; [2]Externalizing Problems; [3]Total problems; [4]Anxious/Depressed; [5]Withdrawn/Depressed; [6]Somatic Complaints; [7]Social Problems; [8]Thought Problems; [9]Attention Problems [10]Rule-Breaking Behavior; [11]Aggressive Behavior; [12]Number/percentage of clinical significant cases ($T > 63/69$) according gender identity norm; [13]Number/percentage of clinical significant cases according at birth assigned sex norm; [14]Significance of McNemar-Test; [15]Odds ratio; [16]95% bias-corrected and accelerated confidence interval.
